# Retinal Expression of Wnt-Pathway Mediated Genes in Low-Density Lipoprotein Receptor-Related Protein 5 (Lrp5) Knockout Mice

**DOI:** 10.1371/journal.pone.0030203

**Published:** 2012-01-17

**Authors:** Jing Chen, Andreas Stahl, Nathan M. Krah, Molly R. Seaward, Jean-Sebastian Joyal, Aimee M. Juan, Colman J. Hatton, Christopher M. Aderman, Roberta J. Dennison, Keirnan L. Willett, Przemyslaw Sapieha, Lois E. H. Smith

**Affiliations:** 1 Department of Ophthalmology, Harvard Medical School, Children's Hospital Boston, Boston, Massachusetts, United States of America; 2 University Eye Hospital Freiburg, Freiburg, Germany; 3 Department of Ophthalmology, Maisonneuve-Rosemont Hospital Research Centre, University of Montreal, Montreal, Canada; The University of Hong Kong, Hong Kong

## Abstract

Mutations in low-density lipoprotein receptor-related protein 5 (Lrp5) impair retinal angiogenesis in patients with familial exudative vitreoretinopathy (FEVR), a rare type of blinding vascular eye disease. The defective retinal vasculature phenotype in human FEVR patients is recapitulated in Lrp5 knockout (*Lrp5^−/−^*) mouse with delayed and incomplete development of retinal vessels. In this study we examined gene expression changes in the developing *Lrp5^−/−^* mouse retina to gain insight into the molecular mechanisms that underlie the pathology of FEVR in humans. Gene expression levels were assessed with an Illumina microarray on total RNA from *Lrp5^−/−^* and WT retinas isolated on postnatal day (P) 8. Regulated genes were confirmed using RT-qPCR analysis. Consistent with a role in vascular development, we identified expression changes in genes involved in cell-cell adhesion, blood vessel morphogenesis and membrane transport in *Lrp5^−/−^* retina compared to WT retina. In particular, tight junction protein claudin5 and amino acid transporter slc38a5 are both highly down-regulated in *Lrp5^−/−^* retina. Similarly, several Wnt ligands including Wnt7b show decreased expression levels. Plasmalemma vesicle associated protein (plvap), an endothelial permeability marker, in contrast, is up-regulated consistent with increased permeability in *Lrp5^−/−^* retinas. Together these data suggest that Lrp5 regulates multiple groups of genes that influence retinal angiogenesis and may contribute to the pathogenesis of FEVR.

## Introduction

Familial exudative vitreoretinopathy (FEVR) is a rare hereditary eye disease with abnormalities in retinal vascular development [1]. Retinal blood vessel development in humans normally starts during the fourth month of gestation and is completed just before birth [2], [3]. Infants with FEVR, in contrast, are born with an incomplete retinal vasculature, leaving the peripheral retina avascular. At the same time, hyaloid vessels, an embryonic ocular vascular bed that normally regresses after birth, persists in FEVR patients [4]. In the most severe cases of FEVR, blindness results from tractional retinal detachments as a consequence of hypoxia-induced neovascularization secondary to incomplete retinal vascularization. Similar abnormalities in retinal vasculature are also observed in patients with X-linked Norrie disease [5], [6].

Genetic studies have linked FEVR, Norrie disease and Coats' disease with mutations in genes encoding components of the Wnt signaling pathway, known to be involved in development and disease [7], [8], [9]. Low-density lipoprotein receptor-related protein 5 (Lrp5), a Wnt co-receptor, is implicated in both autosomal dominant and recessive forms of FEVR [10], [11]. In addition, the Wnt receptor frizzled4 is linked to autosomal dominant FEVR [12], [13], and the Wnt ligand Norrin is associated with Norrie disease, Coats' disease and X-linked recessive FEVR [14], [15], [16]. Canonical Wnt signaling starts with binding of Wnt ligands, a group of small proteins to an activated Wnt receptor complex composed of Frizzled and Lrp5/6. Wnt ligand binding stabilizes β-catenin in the cytoplasm of the activated cell from where it translocates to the nucleus and binds nuclear T-cell factor/lymphoid enhancer factor (TCF/LEF) to control activation of Wnt-responsive genes [8].

The ocular phenotype of human FEVR disease is replicated in Lrp5 deficient mice [17], [18]. In addition, *Lrp5^−/−^* mice also have low bone density and persistent embryonic hyaloid vessels in the eye [17], [18], recapitulating human autosomal-recessive osteoporosis-pseudoglioma syndrome (OPPG), a form of FEVR. In contrast to humans, retinal vasculature in mice develops postnatally which makes the *Lrp5^−/−^* mouse a useful experimental model for studying Wnt signaling and the pathogenesis of FEVR [19], [20]. Similar to human FEVR patients, the retina of *Lrp5^−/−^* mice displays delayed vessel growth in the peripheral retina [21] and lack of deep layers of retinal capillary networks [19], [20]. Inadequate vascularization in the retina contributes to the subsequent formation of hypoxia-driven microaneurysm-like vascular lesions [22], also mimicking those observed in human FEVR patients. As additional proof that the Wnt pathway is important in the pathologic events leading to FEVR, delayed and incomplete retinal vascular development is also observed in *Norrin^−/−^* and *Frizzled4^−/−^* mice [16], [23] which lack other elements of the Wnt pathway.

The purpose of this study is to analyze gene expression changes triggered by the absence of Wnt signaling in *Lrp5^−/−^* mouse retinas. Analysis of gene expression in a mouse model of FEVR is important considering that there is no human retinal expression data available in FEVR patients. Since *Lrp5^−/−^* retinas lack the Lrp5-mediated activation of Wnt-responsive genes, any differentially regulated genes identified between WT and *Lrp5^−/−^* mouse can be potential mediators of Wnt-driven regulation of retinal blood vessel growth. Detailed analysis of these differentially regulated genes therefore has the potential to help elucidate the molecular events leading to the defective retinal vascular phenotype observed in human FEVR patients.

## Results

### Delayed retinal vascular development and persistent hyaloid vessels in Lrp5^−/−^ mice

**Figure 1:**
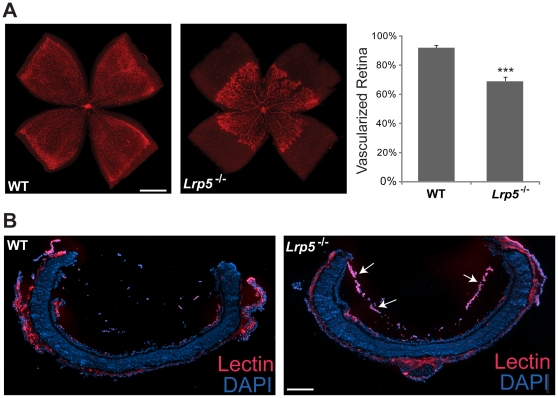
Delayed development of the superficial retinal vasculature and persistent hyaloid vessels in *Lrp5* null mice. (A) Left: retinal whole mounts stained with isolectin B_4_-594 from WT and *Lrp5* null mice at post-natal day (P) 8. Right: quantification of vascularized retinal area in WT and *Lrp5* null mice at P8. n = 5–10 per group, ***p<0.001. (B) Retinal cross sections of WT mice and *Lrp5* null mice at P8 stained for endothelial cells with isolectin B_4_-594 (red) and cell nuclei (DAPI, blue). Arrows indicate persistent hyaloid vessels in *Lrp5* null retina. Scale bars: 500 µm.

Characterization of *Lrp5^−/−^* retinas revealed delayed outgrowth of the superficial capillary network at P8 with retinal vessels covering 69.3±2.7% of the total retinal area, compared with 92.2±1.5% in WT retinas (n = 5–10 per group; *p*≤0.0001, **Fig. 1A**), suggesting that loss of Lrp5 causes delayed retinal vascular growth, similar to that seen in *Norrin^−/−^* and *Frizzled4^−/−^* retinas [21], [23]. As the retinal vasculature develops, hyaloid vessels, an embryonic vascular bed that provides nutrients to the developing lens, normally regress after birth. In *Lrp5^−/−^* eyes, however, persistent hyaloid vessels are seen at P8 compared with WT retina (**Fig. 1B**), suggesting a defect in hyaloid regression in the absence of Lrp5.

### Defective retinal vasculature and lack of deep layer vascular networks in Lrp5^−/−^ retina

**Figure 2:**
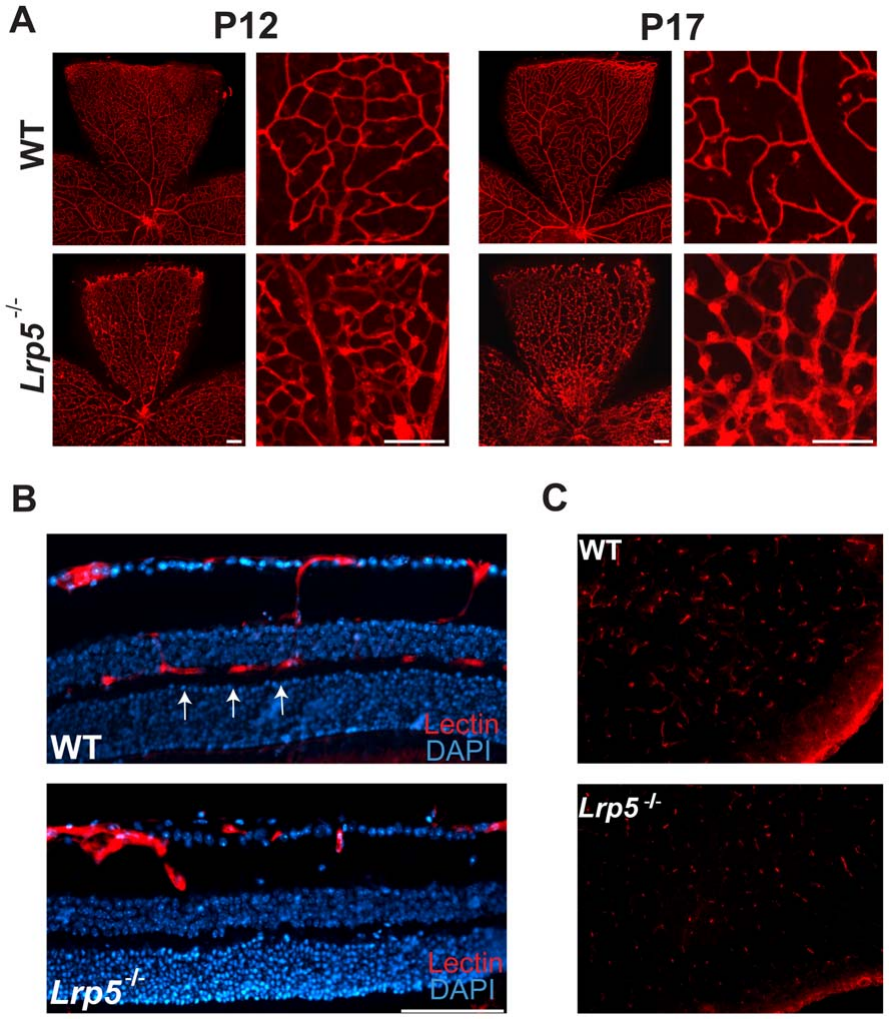
Abnormal vascular development in the inner and outer retina and forebrain of *Lrp5* null mice. (A) Retinal whole mounts of WT and *Lrp5* null mice stained for endothelial cells with isolectin B_4_-594 at P12 and 17. Enlarged images highlight the abnormal vessel growth in the *Lrp5* null retina. (B) Retinal cross sections from P30 WT and *Lrp5* null mice stained for endothelial cells with isolectin B_4_-594 (red) and cell nuclei with DAPI (blue). Arrows indicate deep layers of retinal vasculature which is present in WT retina but absent in *Lrp5* null retina. (C) Cross sections of the forebrain from P8 WT and *Lrp5* null mice with endothelial cells stained with isolectin B_4_-594. Scale bar: 100 µm.

At P12, *Lrp5^−/−^* retinas start to develop dilated vessels with enlarged microaneurysm-like lesions in the superficial layer (**Fig. 2A**). Progression of these lesions is seen at P17 (**Fig. 2A**), and the lesions persist into adulthood [22]. Development of intermediate and deep retinal capillary networks in inner and outer plexiform layers normally begins around P8 and is completed by P21 in WT mice. However, *Lrp5^−/−^* retinas fail to form these deeper layers of capillary vessels, as shown in retinal cross sections of adult mice (**Fig. 2B**). A similar absence of the retinal vascular network has also been reported in homozygous *Lrp5^r18^* mice with frame shift mutation [19], [20], suggesting that Lrp5 is required for sprouting angiogenesis in the deep layers of the retinal capillary bed.

### Decreased vascular density in Lrp5^−/−^ brain

The decreased vascular growth in the retina likely reflects reduced overall vascular density in the central nervous system. We stained brain sections from *Lrp5^−/−^* and WT mice with isolectin to assess brain vessel density. At P8, vascular density in the forebrain is less in *Lrp5^−/−^* mice compared with wild type mice (**Fig. 2C**). Defective CNS angiogenesis is also observed in *Frizzled4^−/−^* mice [16], as well as in *Wnt7a^−/−^* and *Wnt7b^−/−^* mice with disruption of Wnt signaling [24], [25], suggesting that the Wnt signaling pathway is critical for blood vessel growth in the central nervous system. This agrees with the clinical observations in Norrie disease patients with vascular defects in the cerebellum, impaired motor skills and mental retardation [6].

### Expression of Lrp5, Norrin, Frizzled4 and Disheveled during development of wild type retina

**Figure 3:**
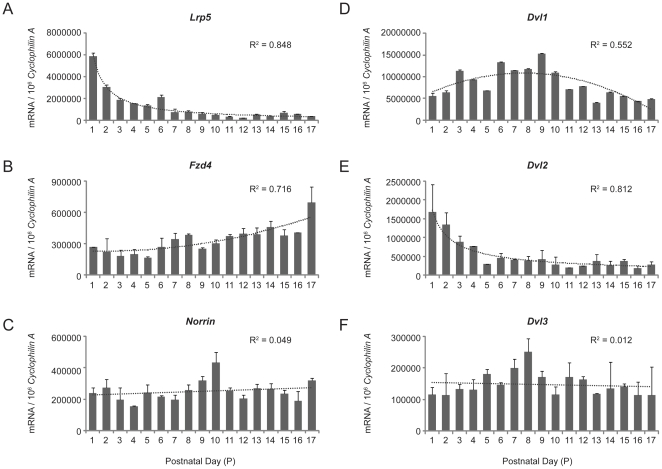
Expression levels of *Lrp5*, *Norrin*, *Frizzled4* and *Dvl* mRNA during retinal development in wild type mice. Quantification of mRNA (A) *Lrp5*, (B) *Norrin*, (C) *Frizzled4*, (D) *Dvl1*, (E) *Dvl2*, and (F) *Dlv3* was performed with RT-qPCR during normal retinal development from P1-P17. Expression levels were normalized against house keeping gene *Cyclophilin A*. Trend lines were fitted with polynomial, linear or power regression.

To understand the temporal expression of Wnt ligands and receptors in the retina, we assessed expression levels of the Wnt ligand Norrin as well as expression of the receptors Frizzled4 and Lrp5 during normal development in wild type mouse retina. From P1 to P17, *Lrp5* mRNA expression decreases (**Fig. 3A**), consistent with a role for Lrp5 during neovessel development as opposed to expression in mature vessels [22]. In contrast, the expression level of Wnt receptor *Frizzled4* tends to increase as the retina develops (**Fig. 3B**). *Norrin* expression levels, on the other hand, remains relatively unchanged from P1 to P17 (**Fig. 3C**). These changes may reflect complex interactions between multiple Wnt ligands and receptors that occur during retinal development. Other Wnt ligands such as Wnt7b, and Frizzled receptors other than Frizzled4, eg. Frizzled5, are also important for vascular and neuronal development in the eye, and may interact with Lrp5 [26], [27]. In addition, Norrin and Frizzled4 may also bind Lrp6, another Wnt co-receptor with essential functions for eye development [28]. Moreover, we also assessed expression of disheveled (Dvl), a cytoplasmatic phospho-protein that is required for Wnt signaling. Dvl2 shows remarkably similar decreasing expression pattern as Lrp5, while as Dvl1 and Dvl3 does not show substantial changes during development (**Figure 3D, E, F**).

### Differential expression of retinal genes in Lrp5^−/−^ eye

**Figure 4:**
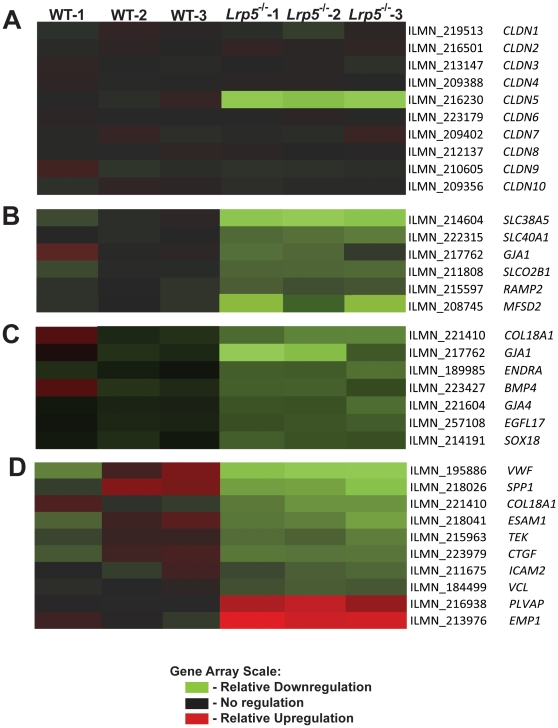
Regulation of tight junction, membrane transport, angiogenic, and cell adhesion genes in the *Lrp5* null retina. Heat maps illustrate the results of a gene array run from whole retinal total mRNA. The most regulated families of genes were (A) claudin family genes, (B) membrane transport genes, (C) angiogenic regulatory genes, and (D) cell adhesion/cell-cell junction genes. Each sample is represented by a block: either wild-type (WT) samples 1 through 3, and Lrp5 null samples 1–3. Relative down-regulation of expression in *Lrp5* null retina compared to WT retina is represented by green, while relative up-regulation is in red. No relative regulation is black (See scale on Figure).

To assess candidate genes differentially expressed in *Lrp5^−/−^* retinas, we examined a gene expression microarray (Illumina mouse-6 expression BeadChip) of P8 *Lrp5^−/−^* retinas and WT control retinas (n = 3 per group). P8 is chosen because the development of deeper retinal vessel networks, defective in *Lrp5^−/−^* retina, begins at P8. Among ∼45,000 probe sequences on the array, 11,790 expressed probes were detected. After initial quality control, background analysis and normalization with Bead Studio software, the array data was further analyzed using the SAM program (Significant Analysis of Microarray) [29]. Using p<0.05 and a 1.3 fold change cutoff between gene expression in *Lrp5^−/−^* compared to WT retinas with an estimated false discovery rate of ∼19%, we identified 80 genes down-regulated and 13 genes up-regulated. These genes were grouped using Gene Ontology and the most significantly regulated gene groups are summarized in **Table 1**. Among the most altered are cell adhesion molecules, membrane transporters, and molecules involved in blood vessel growth and morphogenesis. Tight junction protein claudin5 (Cln5), an endothelial specific protein that plays a critical role in maintaining blood retinal barrier (BRB) [30], is significantly down-regulated about 9 fold in *Lrp5^−/−^* retina. Slc38a5 (solute carrier family 38, member 5), a system N sodium-coupled amino acid transporter, is similarly down-regulated 7 fold. Other mRNAs down-regulated in *Lrp5^−/−^* retinas include major facilitator superfamily domain containing 2 (Mfsd2) (∼3 fold), gap junction membrane channel protein alpha 1 (Gja1) (∼2 fold) and transcription factor Sox18 (∼1.4 fold). Two mRNAs significantly up-regulated in *Lrp5^−/−^* retinas are plasmalemma vesicle associated protein (Plvap), a marker for fenestrated blood vessel associated with increased vascular permeability [31], [32], and epithelial membrane protein 1 (EMP1). To illustrate expression levels of identified genes, heat maps were generated for claudin family proteins, membrane transporters, cell adhesion proteins and blood vessel growth factors (**Fig. 4**). Among 10 members of the claudin family of genes, only Cln5 is significantly regulated in the absence of Lrp5. The down-regulation of tight junction protein Cln5 and increased expression of Plvap are consistent with the observed increased vascular permeability in *Lrp5^−/−^* retinas [20], [22]. It is important to note that several of the most significantly regulated genes (Cln5, Scl38a5, Mfsd2, Plvap) are also similarly regulated in *Norrin^−/−^* retinas [33] (**Table 2**), suggesting the possibility that the related retinal vascular phenotype observed in *Lrp5^−/−^* and *Norrin^−/−^* mice is mediated by common molecular mechanisms in the absence of different Wnt signaling pathway components.

**Table 1:** Selected groups of genes regulated in *Lrp5^−/−^* retina.

Genes:	*Fold change in Lrp5^−/−^ retina*
***Cell Adhesion/Junction***	
claudin 5 (Cldn5)	(−) 8.97
Von Willebrand factor homolog (Vwf)	(−) 2.56
secreted phosphoprotein 1 (Spp1)	(−) 2.32
CD93 antigen (CD93)	(−) 1.89
procollagen, type XVIII, alpha 1 (Col18a1)	(−) 1.72
endothelial cell-specific adhesion molecule (Esam1)	(−) 1.73
endothelial-specific receptor tyrosine kinase (Tek)	(−) 1.48
connective tissue growth factor (Ctgf)	(−) 1.55
intercellular adhesion molecule 2 (Icam2)	(−) 1.31
vinculin (Vcl)	(−) 1.29
plasmalemma vesicle associated protein (Plvap)	(+) 1.78
epithelial membrane protein 1 (EMP1)	(+) 2.08
***Membrane Transporter***	
solute carrier family 38, member 5 (Slc38a5)	(−) 7.11
major facilitator superfamily domain containing 2 (Mfsd2)	(−) 3.26
solute carrier family 40 (iron-regulated transporter), member 1 (Slc40a1)	(−) 2.22
gap junction membrane channel protein alpha 1 (Gja1)	(−) 2.05
solute carrier family 3, member 4 (Slc3a4)	(−) 2.01
solute carrier organic anion transporter family, member 2b1 (Slco2b1)	(−) 1.92
receptor (calcitonin) activity modifying protein 2 (Ramp2)	(−) 1.53
***Blood vessel development/morphogenesis***	
gap junction membrane channel protein alpha 1 (Gja1)	(−) 2.05
procollagen, type XVIII, alpha 1 (Col18a1)	(−) 1.72
endothelin receptor type A (Ednra)	(−) 1.49
bone morphogenetic protein 4 (Bmp4)	(−) 1.42
gap junction protein, alpha 4 (Gja4)	(−) 1.42
EGF-like domain 7 (Egfl7)	(−) 1.38
SRY-box containing gene 18 (Sox18)	(−) 1.38

Note: Retinas were isolated from P8 *Lrp5^−/−^* mice and age matched WT control mice. RNA was isolated and assessed with Illumina gene expression microarray. (−) indicates decreased expression in *Lrp5^−/−^* retina and (+) indicates increased expression in *Lrp5^−/−^* retina compared to WT control.

**Table 2:** Common genes regulated in *Lrp5^−/−^* and *Norrin^−/−^* retinas.

	Fold change	
Genes:	*Lrp5^−/−^*	*Norrin^−/−^* *
***Decreased***		
claudin 5 (Cldn5)	(−) 8.97	(−) 2.03
solute carrier family 38, member 5 (Slc38a5)	(−) 7.11	(−) 14.03
major facilitator superfamily domain containing 2 (Mfsd2)	(−) 3.26	(−) 2.44
EGF, latrophilin seven transmembrane domain containing 1 (Eltd1)	(−) 2.27	(−) 1.72
Apolipoprotein D (Apod)	(−) 1.67	(−) 2.06
Angiotensin receptor-like 1 (Agtrl1)	(−) 1.40	(−) 1.92
Adenomatosis polyposis coli down-regulated 1 (Apcdd1)	(−) 1.25	(−) 1.66
***Increased***		
plasmalemma vesicle associated protein (Plvap)	(+) 1.78	(+) 1.86
Adrenomedullin (Adm)	(+) 1.37	(+) 2.03

Note: Retinas were isolated from P8 *Lrp5^−/−^* mice and age matched WT control mice. RNA was isolated and assessed with Illumina gene expression microarray. (−) indicates decreased expression in *Lrp5^−/−^* retina compared to WT retina, and (+) indicates increased expression in *Lrp5^−/−^* retina.

*: Genes regulated in P7 *Norrin^−/−^* retina were adapted from Schafer et. al. IOVS, 2009 [33].

### Validation of selected genes with RT-qPCR

**Figure 5:**
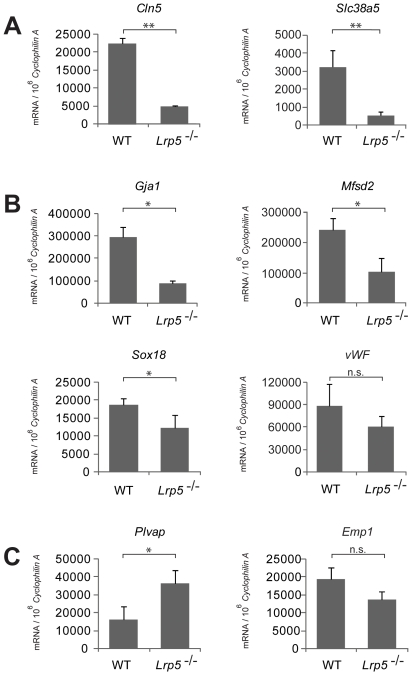
Confirmation of gene expression differentially regulated in Lrp5 null retina with RT-qPCR. Quantification of mRNA (A) *Cln5* and *Slc38a5*, (B) *Gja1*, *Msfd2*, *Sox18*, and *vWF*, and (C) *Plvap* and *EMP1* in WT and *Lrp5* null retina with RT-qPCR at P8. Expression levels were normalized against housekeeping gene *Cyclophilin A*. *p<0.05, **p<0.01.

To validate the genes identified with microarray analysis, we performed RT-qPCR to assess mRNA expression level of selected genes. This analysis confirmed Cln5 and Slc38a5 mRNA levels to be significantly down-regulated in *Lrp5^−/−^* retinas, by 5 fold and 6 fold respectively (**Fig. 5A**). Significant down-regulation was also verified for Gja1 (∼3 fold), Mfsd2 (∼5 fold) using RT-qPCR (**Fig. 5B**). Transcription factor Sox18 is significantly down-regulated on qPCR (∼1.5 fold), with vWF showing a trend of down –regulation (1.4 fold) (**Fig. 5B**). Similarly, qPCR analysis validated increased expression of Plvap (∼4 fold) in *Lrp5^−/−^* retinas (**Fig. 5C**). The increased regulation of EMP1 can not be confirmed with RT-qPCR, suggesting that EMP1 is likely one of the false positive discovery from gene array. Together these data confirm differential expression of the top candidate genes identified with microarray in *Lrp5^−/−^* retinas. Expression levels of these genes are likely regulated either directly as transcription targets of Wnt signaling in cells that normally express LRP5, or through secondary effects responding to primary changes in retinal vascular and neuronal defects in the absence of Lrp5.

### Regulation of Wnt ligands in Lrp5^−/−^ retina

**Figure 6:**
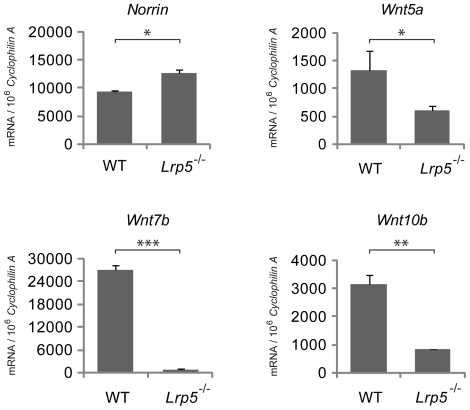
Wnt ligands and receptor regulated in P8 *Lrp5* null retina. Wnt ligand *Norrin*, Wnt5a, Wnt7b, and Wnt10b mRNA expression in WT and *Lrp5^−/−^* whole retinas were quantified with RT-qPCR and normalized to *Cyclophilin A* expression. *p<0.05, **p<0.01.

To assess whether components of the Wnt pathway are regulated in *Lrp5^−/−^* retinas, we assessed Wnt ligands, receptors and other downstream effectors in gene array and found no substantial changes (**Fig. S1**). In order to corroborate these findings, we screened with RT-qPCR expression levels of several Wnt ligands in *Lrp5^−/−^* and WT retinas at P8. Interestingly we found Norrin levels to be slightly increased in *Lrp5^−/−^* retinas compared to WT (**Fig. 6**). In contrast, the ligands Wnt5a and Wnt10b are both significantly down-regulated by 2–4 fold in *Lrp5^−/−^* retinas compared to WT (**Fig. 6**). In particular, Wnt7b level is significantly down-regulated by >10 fold (**Fig. 6**). Wnt7b secreted by macrophages is implicated in regulation of hyaloid regression [26]; hence decreased Wnt7b levels are likely associated with or contribute to the observed persistence of hyaloid vessels in *Lrp5^−/−^* eyes.

## Discussion

In this study we assessed gene expression changes in *Lrp5^−/−^* retinas, which may contribute to the delayed and incomplete retinal vascular development in the absence of Lrp5. We found *Lrp5^−/−^* retinas have delayed growth of the superficial layer of retinal vessel and persistant hyaloid vessels. *Lrp5^−/−^* retinas also fail to develop deep layers of retinal capillary networks. As the retina grows and becomes metabolically active, tissue ischemia and hypoxia occur, resulting in abnormal aggregative endothelial lesions in the superficial layer staring from P12 and persisting into adulthood. These cellular changes correspond to visual function deficiency in Lrp5 loss of function mutant mice [19]. Previous studies have reported presence of Lrp5 in retinal vascular endothelial cells and Muller cells [19], [22], suggesting these cells are likely most impacted by lack of Lrp5 to cause the defective retinal vascular and functional phenotype in *Lrp5^−/−^* mice.

The most significantly regulated genes found in *Lrp5^−/−^* retina include cell adhesion proteins, membrane transporters and genes involved in blood vessel growth and morphogenesis. Of particular interest is the tight junction protein Cln5 which is down-regulated in *Lrp5^−/−^* retinas approximately 9 fold. Previous studies have found that Cln5 is regulated in a Wnt dependent manner via ß-catenin [22], [34]. Regulation of Cln5 by Wnt signaling is likely mediated by transcription factor Sox18 [22] which is also significantly down-regulated in the *Lrp5^−/−^* retina. A previous study from our group found that blocking Cln5 significantly suppresses blood vessel endothelial cell growth [22], suggesting that the defective vascular phenotype of *Lrp5^−/−^* retinas is likely attributable in part to Cln5 deficiency. As an integral part of tight junctions, Cln5 may be essential for endothelial cell adhesion, tube formation and organized migration of endothelial cells. Importantly in this context, Cln5 is also significantly down-regulated in *Norrin^−/−^* retinas to a similar extent [33], raising the possibility that Cln5 may mediate common molecular events underlying similar retinal vascular defects in the absence of Wnt signaling.

We found that Slc38a5, an amino acid transporter expressed mainly in Muller cells, ganglion cells and endothelial cells [19], [35], [36], is also significantly down-regulated in *Lrp5* ^−/−^ retinas confirming previous reports. Interestingly, Slc38a5 is down-regulated to a similar extent in *Norrin^−/−^* retinas [33]. Slc38a5 is a neutral amino acid transporter responsible mostly for glutamine uptake in the retina. It is not yet clear whether the *Lrp5* ^−/−^ retina has a deficiency in glutamine transport or glutamate synthesis. However, *Lrp5^r18^* mutant mice have reduced b-wave ERG responses, which is likely attributed to vascular deficiency in the inner retina and may be associated with loss of the glutamate transporter [19], [37]. Whether down-regulation of slc38a5 is linked to the impaired retinal vascular development and neuronal function in Lrp5^−/−^ retina needs further investigation.

Plvap, a vascular permeability marker, is highly up-regulated in *Lrp5* ^−/−^ retina, likely reflecting increased breakdown of the blood retinal barrier [20], [22]. Loss of Wnt signaling is associated with decreased blood barrier integrity not only in the retina but also in the central nervous system in the absence of Wnt ligands Wnt7a and Wnt7b [24], [25]. Similarly, loss of Frizzled4 also causes impairment in blood brain barrier function and increased vascular leakage [16], [21]. In *Lrp5* ^−/−^ retinas, the observed BRB breakdown may be precipitated by decreased levels of Cln5, an essential component of tight junctions [30]. This is consistent with the observation of impaired blood brain barrier function in *Cln5* ^−/−^ mice [38].

The genes identified in this study may not only help to understand the abnormal retinal vascular development in *Lrp5* ^−/−^ eyes, but also elucidate the molecular basis of persistent hyaloid vessels observed in *Lrp5* ^−/−^ eyes. Expression levels of Wnt7b, which has been implicated in macrophage-mediated hyaloid vessel regression [26], is found to be significantly down-regulated in *Lrp5* ^−/−^ eyes. Hence, persistent hyaloid vessels in *Lrp5* ^−/−^ eyes may be partially attributable to Wnt7b deficiency. Persistent hyaloid vessels often occur in association with delayed retinal vascular development in both humans and mice. It remains, however, debatable which process, hyaloid regression or delayed retinal vessel growth, is the primary event. It is conceivable that inadequate retinal vascular growth can cause retinal hypoxia which impedes regression of hyaloid vessels [39]. On the other hand, persistent hyaloid vessels can also suppress the hypothesized “physiologic hypoxia” required for normal retinal vascular development [40], [41]. Interestingly, mice with conditional depletion of VHL (von Hippel-Lindau tumor suppressor protein), which is essential for HIF (hypoxia-inducible factor) signaling, develop persistent hyaloid vasculature [42]. This phenotype is similar to mice with loss of Wnt signaling. Recently a study showed HIF1α can negatively regulate Wnt/ß-catenin activity [43]. Together these data suggested oxygen-sensing mechanisms likely play a significant role in the process of hyaloid regression and retinal vessel development, and Wnt signaling may act synergistically with oxygen-sensing pathways to mediate both hyaloid vessel regression and retinal vascular development.

As Wnt signaling is implicated in many biological processes during development and disease, it is sensible to question whether Wnt signaling acts beyond the hereditary retinopathies to play a role in other more prevalent, postnatally occurring retinopathies such as retinopathy of prematurity (ROP) and diabetic retinopathy. Our previous work indicates that Wnt signaling affects not only vascular growth during retinal development but also pathologic neovascularization in proliferative ischemic retinopathy [22]. Studies from other groups also show that Wnt signaling affects inflammation and oxidative stress in animal models of diabetic retinopathy [44], [45], [46] and age-related macular degeneration [47]. Targeting Wnt signaling pathway, therefore, appears to be an appealing approach to prevent and treat not only genetic eye disorders such as FEVR, but also other vascular eye diseases.

## Materials and Methods

### Animals

These studies adhered to the Association for Research in Vision and Ophthalmology (ARVO) Statement for the Use of Animals in Ophthalmic and Vision Research and were approved by the Children's Hospital Boston Animal Care and Use Committee (animal protocol approval ID 10-08-1770R). *Lrp5^−/−^* mice (stock no. 005823; Jackson Laboratory, developed by Deltagen Inc.) and its wild type control C57Bl/6J mice (stock no. 000664; Jackson Laboratory) were used for this study.

### Retina dissection, vessel staining and flat mount

Mice at postnatal day 8, 12 and 17 were anesthetized with Avertin (Sigma-Aldrich) and sacrificed by cervical dislocation. Eyes were enucleated and fixed in 4% paraformaldehyde for 1 h at room temperature, followed by isolation and dissection of the retina. To visualize the retinal vasculature, retinas were stained overnight at room temperature with fluoresceinated *Griffonia Bandeiraea Simplicifolia* Isolectin B_4_ (Alexa Fluor 594 conjugated; I21413; Invitrogen; 1∶100 dilution) in 1 mM CaCl_2_ in PBS. After 2 h of washes in PBS, retinas were whole-mounted onto Superfrost/Plus microscope slides (12-550-15; Fisher Scientific) with the photoreceptor side down. Retinas were then imbedded in SlowFade Antifade reagent (S2828; Invitrogen) to preserve the stain. Retinal vasculatures were imaged with a Zeiss microscope. Vascularized retinal areas were quantified with photoshop as described previously [48], [49], [50].

### Eye and brain sectioning and staining

For histochemistry, eyes and brains were enucleated and fixed in 4% paraformaldehyde for 1 h at room temperature, followed by removal of cornea and lens. The eye cups were subsequently rinsed with PBS and placed in 30% sucrose overnight at 4°C, embedded in OCT and frozen on dry ice. Retinal samples were cryosectioned into 12-µm sections, and collected on slides. Sections were stained with isolectin (1∶100 in PBS with 1 mM CaCl_2_) to visualize vessels and mounted with Vectashield mounting medium for fluorescence with DAPI (Vector Laboratories, Inc. Burlingame, CA).

### RNA isolation and cDNA preparation

For each time point, total RNA was extracted from the retinas of 6 mice, each from a different litter. Retinas were lysed with a mortar and pestle and filtered through QiaShredder columns (Qiagen, Chatsworth, MD, USA). RNA was then extracted as per manufacturer's instructions using the RNeasy Kit (Qiagen). The RNA was then pooled to reduce biologic variability (n = 6). To generate cDNA, 1 µg total RNA was treated with DNase I (Ambion Inc.) to remove any contaminating genomic DNA, and was then reverse transcribed using random hexamers, and SuperScript III reverse transcriptase (Invitrogen Corp., Carlsbad, CA, USA).

### Gene expression microarray

Total retinal RNA was isolated from 3 *Lrp5* ^−/−^ and 3 WT mice. Gene expression microarray analysis was performed using an Illumina mouse gene microarray, with each sample being a biological replicate (n = 3 per group; Mouse-WG6 expression BeadChip; Illumina, San Diego, CA). All data is MIAME compliant and the raw data was deposited in a MIAME compliant database (GEO, accession number GSE32145). The chip contains ∼45,000 probe sets representing ∼34,000 genes. Microarray studies, from cDNA synthesis to raw data normalization were performed by the Molecular Genetics Core Facility at Children's Hospital Boston. Briefly, total RNA (1 µg each) were reverse transcribed, followed by a single *in vitro* transcription amplification to incorporate biotin-labeled nucleotide, and subsequent hybridization and staining with strepatavidin-Cy3 according to the manufacturer's instructions. The chip was scanned with Illumina BeadArray Reader to measure the signal intensity by the labeled target. Raw data were analyzed with the microarray software (Bead Studio Gene Expression version 3.4.0) for quality control, background analysis and normalization with rank invariant algorithm. Normalized data were further analyzed with the SAM program (Significant Analysis of Microarray) [29] using p<0.05 and a delta of 0.19. Resulting gene lists for both *Lrp5^−/−^* and WT retinas were grouped using online tool Gene Ontology Enrichment Analysis Software (GOEAST Tools), which is hosted by Institute of Genetics and Developmental Biology, Chinese Academy of Sciences [51]. Heat maps demonstrating differential gene expression were generated by adjusting the average signal of each sample to their respective log10 values. The average of the three WT signals was then used as the baseline for normal gene expression. Each value from *Lrp5* ^−/−^ retina was normalized to the average WT value and assigned a number between −1, which represented downregulation, 0, which represented no gene regulation, and +1, which represented upregulation. These values were then plotted in Microsoft Excel and colors were assigned to values using the Conditional Formatting function. Maps were then imported into Adobe Illustrator (CS4) to enhance contrast and resolution.

### Quantitative real-time PCR analysis of gene expression

PCR primers targeting *Lrp5* (F: 5′-AAG GGT GCT GTG TAC TGG AC-3′, R: 5-AGA AGA GAA CCT TAC GGG ACG-3′), *Frizzled4* (F: 5′-TTC CTT TGT TCG GTT TAT GTG CC-3′, R: 5′-CTC TCA GGA CTG GTT CAC AGC-3′), *Norrin* (F: 5′-GTG AGG GGC ACT GCA GCC AG-3′; R: 5′-CAG CGC AGA CGC AGA GCC TT-3′), *Claudin5* (F: 5′-GCA AGG TGT ATG AAT CTG TGC T-3′, R: 5′-GTC AAG GTA ACA AAG AGT GCC A-3′), *Plvap* (F: 5′-GCT GGT ACT ACC TGC GCT ATT-3′, R: 5′-CCT GTG AGG CAG ATA GTC CA-3′), *Wnt5a* (F: 5′-CAA CTG GCA GGA CTT TCT CAA-3′, R: 5′-CAT CTC CGA TGC CGG AAC T-3′), *Wnt7b* (F: 5′-GGT GTG GCA GTG TAC CTG CAA-3′, R: 5′-GTG AAG ACC TCG GTG CGC T-3′), *Wnt10b* (F: 5′-GAAGGGTAGTGGTGAGCAAGA-3′, R: 5′-GGT TAC AGC CAC CCC ATT CC-3′), *Gja1* (F: 5′- ACA GCG GTT GAG TCA GCT TG-3′, R: 5′- GAG AGA TGG GGA AGG ACT TGT -3′), *EMP1* (F: 5′- TTG GTG CTA CTG GCT GGT CT -3′, R: 5′- CAT TGC CGT AGG ACA GGG AG-3′), *Mfsd2* (F: 5′- AGA AGC AGC AAC TGT CCA TTT -3′, R: 5′- CTC GGC CCA CAA AAA GGA TAA T-3′), *Sox18* (F: 5′- ATG CCA CTA CAC TCC CCT ACC A-3′, R: 5′- CTG CTC TCT TCT GGA CAG GAC AT-3′), *slc38a5* (F: 5′- CAA CCT CAG CAA CGC TAT CAT-3′, R: 5′- CAG GTC CAA ATG CCC TCT G-3′), *Dvl1* (F: 5′- ATG AGG AGG ACA ATA CGA GCC-3′, R: 5′- GCT TCC GAA CTA GCC GAG AG-3′), *Dvl2* (F: 5′- TGT CGT CAG ATA CCC CAC AG-3′, R: 5′- CTG GAT ACA TTA GGG TGG AAG GA-3′), *Dvl3* (F: 5′- ACA CGG AGA CCG ACT CCT T-3′, R: 5′- AGG GTA GAT GAA CTG TCA TAG CC-3′), *vWF* (F: 5′- CAA TGG CAC CGT AAC GCA G-3′, R: 5′- TGG AGA GCT TAT AGT ACC CAG C-3′) and an house keeping control gene, *Cyclophilin A* (F: 5′-AGG TGG AGA GCA CCA AGA CAG A-3′, R: 5′-TGC CGG AGT CGA CAA TGA T-3′), were designed using Primer Bank and NCBI Primer Blast Software.Express software (Applied BioSystems). We used three methods to analyze primer sequences for specificity of gene detection. First, NCBI Blast module was used to identify primer and probe sequences that specifically detected the sequence of choice. Second, amplicons generated during a PCR reaction were analyzed using the first derivative primer melting curve software supplied by Applied BioSystems. This analysis determines the presence of amplicons on the basis of a specific melting point temperature. Third, amplicons generated were gel purified and sequenced by the Children's Hospital Boston Core Sequencing Facility. This further confirmed the selection of the desired sequence. Quantitative analysis of gene expression was determined using an ABI Prism 7700 Sequence Detection System (TaqMan) and the SYBR Green Master mix kit. Standard curves for each gene were plotted with quantified cDNA template during each real-time PCR reaction. Each target gene cDNA copy number was normalized to 10^6^ copies of the house keeping gene, *cyclophilin A* using delta-delta CT method.

### Statistics

Results are presented as mean±SEM for animal studies and mean±SD for the non-animal studies. A 2-sample *t* test was used as a post test unless otherwise indicated.

## Supporting Information

Figure S1 Heat maps illustrating expression profiles of other members of the Wnt pathway regulated in *Lrp5* null retina in a gene array run from whole retinal mRNA. The genes analyzed include (A) Wnt ligands, (B) Wnt receptors, and (C) other components downstream of Wnt signaling.
(TIF)
