# Supplementary figures and images for: Retinal Expression of Wnt-Pathway Mediated Genes in Low-Density Lipoprotein Receptor-Related Protein 5 (Lrp5) Knockout Mice
Source: PLoS One. 2012 Jan 17;7(1):e30203. doi: 10.1371/journal.pone.0030203 (PMC3260226; doi:10.1371/journal.pone.0030203)

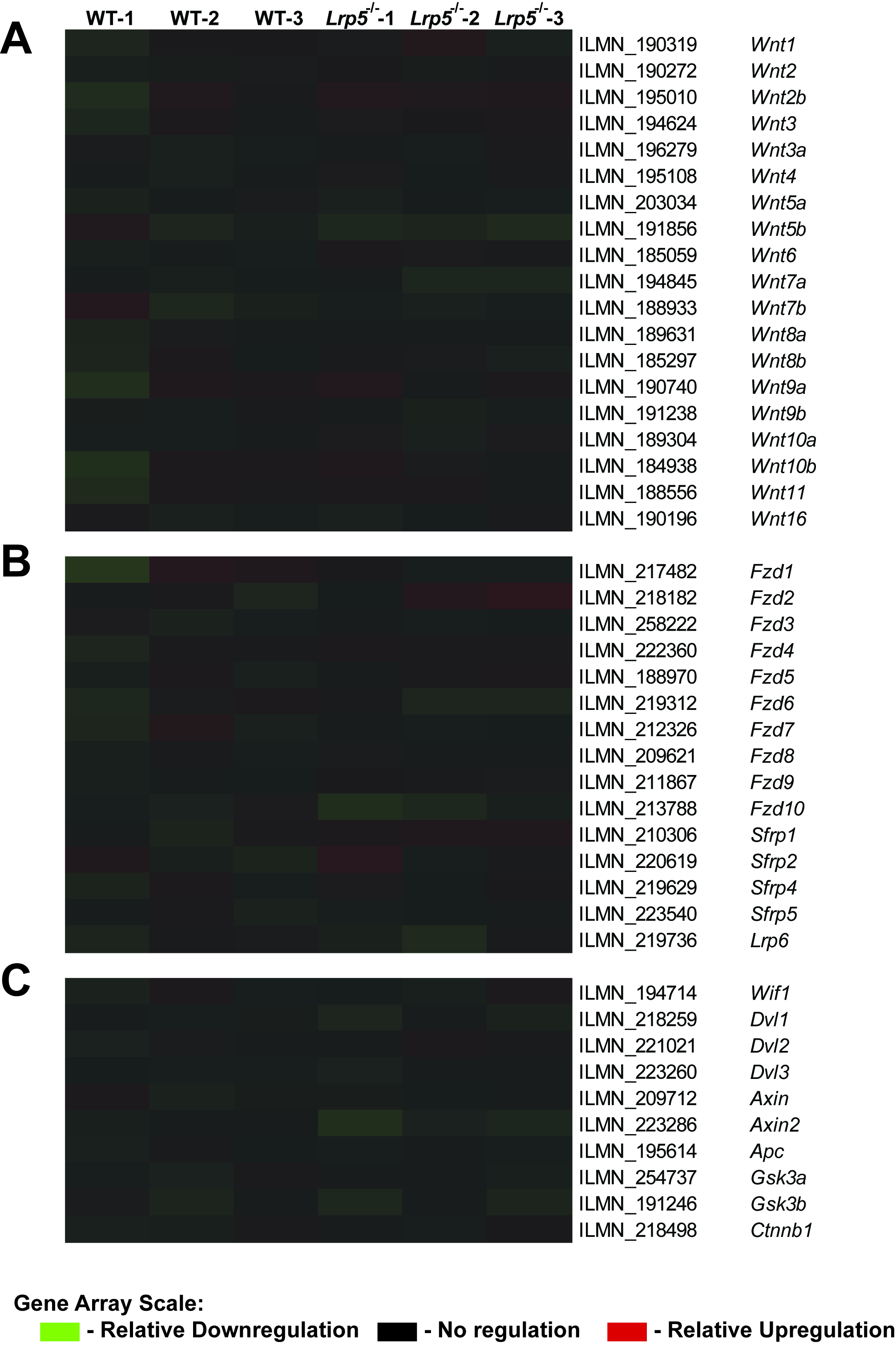

Supplement: Figure S1 — Heat maps illustrating expression profiles of other members of the Wnt pathway regulated in Lrp5 null retina in a gene array run from whole retinal mRNA. The genes analyzed include (A) Wnt ligands, (B) Wnt receptors, and (C) other components downstream of Wnt signaling. (TIF) [file pone.0030203.s001.tif]
